# Evaluation of the knowledge and practice of family physicians in the management of diabetes mellitus type 2 in Iran

**DOI:** 10.1186/s12875-023-02183-6

**Published:** 2023-10-28

**Authors:** Hourvash Haghighinejad, Fatemeh Malekpour, Parisa Jooya

**Affiliations:** Department of Family Medicine, Shiraz Medical School, Shiraz University of Medical Sciences, Shiraz, Iran

**Keywords:** Diabetes Mellitus type 2, Family physicians, Knowledge, Practice

## Abstract

**Background:**

The increasing prevalence and occurrence of type 2 diabetes has made it a widespread epidemic. Being the first line of care, family doctors can play an essential role in this field. The knowledge of these doctors about how to deal with the prevention, diagnosis, and correct treatment of patients is fundamental in reducing the burden of this disease in the community. In this study, we decided to evaluate the knowledge and practice of family doctors in Shiraz-Iran and its related factors in managing Diabetes.

**Method:**

This analytical cross-sectional study was conducted among family doctors of two primary healthcare centers, Shahadai Wal-Fajr Health Center and the Enghlab Health Center in Shiraz, Iran, from March 2021 to August 2021. A researcher-designed diabetes questionnaire consisting of 21 items and a data collection form including demographic information and other related factors was used in this study. An interviewer asked the questions from participants at their workplace and completed the questionnaires. The data were analyzed by SPSS-20 software. A linear regression test was used to investigate the factors affecting the questionnaire score. A one-way ANOVA test was used to compare questionnaire scores among multiple groups.

**Results:**

On average, the participants obtained 62.5% of the total score. The average scores for each question in the screening, the diagnosis, and the treatment sections were 0.5 ± 0.28, 0.65 ± 0.2, and 0.66 ± 0.17, respectively. Physicians’ knowledge about the blood sugar threshold for diagnosing Diabetes was suboptimal, and 81.9, 47, 43 correctly mentioned the FBS, 2hrpp BS, and HbA1c threshold, respectively. Although 95% knew the first line medication but 33.6% prescribed 2nd or 3rd medication for DM treatment. Only 43% knew the goal of therapy. Sixty-three doctors (42%) have not registered any referrals for newly diagnosed uncomplicated diabetic patients, and 37.6% referred these new DM cases to an internist or endocrinologist at the first visit. Microvascular complication screening, such as testing for microalbuminuria and ophthalmologist consultation reported by 32. 89% and 8% of physicians, respectively. Years since graduation was the determining factor of the knowledge level of doctors in this study. Regarding the preferred education method, most participants selected the workshop method as the preferred training method. Virtual education was ranked as the second preferred educational method.

**Conclusion:**

The knowledge and practice of general family doctors were lower than the optimal level in diabetes screening, diagnosis, and treatment. In the treatment of patients, the knowledge of most of the physicians was appropriate in the early stages of treatment, such as determining the time to start the medication and the first line of treatment, but in the follow-up and more advanced treatment, the knowledge and performance of the doctors were less than expected. They prefer to refer patients to higher levels in the healthcare system. Recently graduated physicians had better knowledge and approach to DM management. Therefore, effective periodic training should be conducted as soon as possible to address this pitfall and improve the quality of care. Workshops and virtual education were the most preferred education methods from the participants’ points of view. So, it is suggested that these methods be used as the first training methods. Implementing the specialty training program for family medicine (which has been started in our country for a few years) is the best final solution. In addition, A clinical guideline should be designed for family physicians highlighting these physicians’ roles in the management of Diabetes.

## Background

The increasing prevalence and occurrence of Diabetes mellitus type 2 (DM II) make it a widespread epidemic at the global level [1]. International diabetic federation (IDF) has reported that 536.6 million diabetic patients were living worldwide in 2021. This number will increase to 643 million in 2030 if no preventive action occurs [2].

This problem is growing due to aging, urbanization, lack of activity, and widespread obesity. Diabetes is more prevalent in developing countries, and 75% of people with Diabetes live in low- and middle-income countries [2]. Prevalence of Diabetes mellitus and pre-diabetes in Iranian adults were 18.22 and 10%, respectively, in 2021 [3]. In addition to the known cases of Diabetes, about 20% of people with Diabetes in Iranian society remain undetected [4].

Large-scale studies such as the “Diabetes Control and Complications Trial” (DCCT) and the “UK Prospective Diabetes Study” (UKPDS) have shown that controlling blood sugar, cholesterol, and blood pressure can lead to a reduction in serious complications of Diabetes such as cardiovascular, renal and diabetic neuropathy complications [5, 6]. However, blood sugar control in Iranian society has only happened in 41.2% of diabetic patients who have received treatment [4].

The lack of control over DM management depends on factors related to the patients and the doctors. From primary care physicians’ points of view, the lack of knowledge and skills of these doctors in managing the disease, excessive workload due to multiple assigned tasks, and communication skills have been the main obstacles to reducing the quality of care [7].

Family doctors, as the first line of patient contact with the healthcare system, can play an essential role in this field. Several studies have been conducted in different countries to measure family doctors’ knowledge and performance in type 2 diabetes. A study in Nigeria showed that knowledge of primary care physicians about Diabetes was unacceptable. The need for training and improving physicians knowledge in various aspects of diagnosis, treatment, and prevention has been strongly emphasized [8].

A study concluded that 60% of doctors did not have enough knowledge about the recommended diet for diabetic patients [9].

Another study has investigated DM management by rural family doctors in northern Iran. The study showed a significant shortcoming in physician clinical performance to achieve the goals of DM treatment [10].

The level of physicians’ knowledge is influenced by various factors such as level of education, access and use of evidence-based clinical guidelines, and continuing medical education after graduation.

Family doctors’ education level, which is one of their knowledge-determining factors, varies among countries. In some countries, such as the United States, these physicians complete a specialized course in family medicine. In England, this role is assigned to a doctor, after almost nine years of medical education [11]. General family doctors in Iran provide services to the population under their care after a 7-year general medical education.

Another factor that influences physicians’ knowledge is the application of clinical guidelines. A study in the United States showed that the measurement of HbA1c and blood lipids in diabetic patients, according to the recommended guidelines, were associated with less hospitalization due to renal, vascular, and other diabetes-related complications [12].

A study in Riyadh showed that doctors’ information about the updated diabetes guidelines was insufficient, leading to inadequate knowledge and poor management of the disease [13].

In 2015 a study was conducted in Iran among general practitioners other than family physicians, which showed that the knowledge of these doctors in the field of Diabetes was insufficient. Also, the continuous education programs in this regard did not have a significant effect on increasing the knowledge and clinical performance of these doctors. It has also been shown in this study that the knowledge of doctors about the amount and duration of physical activity required in diabetic patients has no relation with continuing medical education (CME) [14].

Since there has not been a study on the level of knowledge and practice of urban family physicians in Iran regarding the management of Diabetes, we aimed to address this gap in Shiraz, a city in the south of Iran. We also aimed to find out factors related to their knowledge. Also, this study tried to find the preferred educational method from the participants’ points of view to suggest the most effective educational technique.

## Method

This analytical cross-sectional study was conducted among general family doctors of two primary health centers, Shahadai Wal-Fajr Health Center and the Enghelab Health Center in Shiraz, Iran, from March 2021 to August 2021. General family physicians in Iran are medical doctors who complete a 7-year general practitioner program and voluntarily register with the Office of Vice Chancellor of Health as family physicians. The sample size was calculated by using the formula for estimation of single proportion (n = z2 p(1-p)/d2), where P = 0.7 based on previous studies [12], d = 0.15p, with an estimated response rate of 70%. Because of random cluster sampling, this number was multiplied by 1.5 (effect size), so the final sample size was calculated to be 170.

At the time of the study, the total number of working family doctors serving these two Shiraz centers was 722. Cluster sampling was used to enroll physicians in the study. The list of family physicians was requested from Shiraz University of Medical Sciences and sorted by their working place. Ten municipal areas covered by each health center were randomly selected. Family doctors working in those areas were randomly selected from the list.

A professional interviewer guided by one of the researchers referred to the family physicians’ workplace and asked the items of the researcher-made questionnaire. Each interview session lasted about 20 min. The participants also completed a data collection form which took about 10 min. If the physicians did not agree to participate in the study, he/she would be replaced by the next physicians on the list. Physicians who could not complete the questionnaire due to their workload during the session were excluded from the study.

The data collection form consisted of demographic information and other presumably related factors. These factors included the type of employment (government, private), years of graduation, the average number of patients visited per day, years of experience as a family physician, receiving diabetes education after graduation, access to the internet in the workplace, awareness of national guidelines on DM, and studying the national guideline.

### Questionnaire

The questionnaire was designed by a community and family medicine specialist who was a faculty member of the family medicine department, based on previous studies [13, 15] and national diabetes guidelines [16]. The diabetes questionnaire consists of two sections and 21 items. The first section consisting of thirteen multiple-choice questions and three yes and no questions examined knowledge about screening (3 questions), diagnosis ( 8 questions), and treatment (5 questions). Each correct answer would be worth one point. The score of the questionnaire was calculated based on the first part. Therefore the total score was 16. In the second section, six descriptive questions evaluated the participants’ practice norms regarding treatment and patients’ follow-up. This section was not included in the scoring system.

The content validity index (CVI) and content validity ratio (CVR) were used to evaluate the content validity of the questionnaire. For this purpose, the questionnaire was rated by five independent faculty members of the family medicine department, including two internists, one pediatrician, two community specialists, and one family medicine specialist. The content validity index was measured based on experts’ opinions about “relevance”, “clarity”, “simplicity”, and “ambiguity” by a 4-point Likert scoring. To measure the relevance of each item, the experts considered a score from 1 “not relevant”, 2 “requires some corrections”, 3 “relevant but needs minor corrections”, to 4 “completely relevant”. The simplicity of the item was also measured by a score from 1 “not simple”, 2 “needs some corrections”, 3 “is simple but needs minor corrections”, to 4 “is completely simple”. Clarity rating was measured from 1 “clear”, 2 “needs some corrections”, 3 “is clear but needs minor corrections”, to 4 “is completely clear”. Ambiguity took grade from 1 “It is ambiguous”, 2 “It needs some corrections”, 3 “It is not ambiguous but needs minor revision” to 4 “Clear”. As a general rule, the minimum acceptable value for the CVI index is 0.79. If the CVI index of each item were lower than the value, that item would be changed. To determine the CVR, experts examined each item based on the three-point Likert scoring: it is necessary, useful but not necessary, and unnecessary. Then CVR was calculated and interpreted according to the Lawshe Table [17]. After repeated corrections, the content validity ratio became above 95%, and the content validity index for all questions was equal to or more than 80%, which is an acceptable value for content validity. Then, a pilot study was conducted on 29 family doctors to determine the reliability, and Cronbach’s alpha index was calculated as 0.72.

Finally, the data were entered and analyzed in SPSS20 software. Mean, standard deviation, and graphs were used for descriptive analysis. Regression analysis was used to investigate the factors affecting the questionnaire score. One-way ANOVA was used to compare the questionnaire scores among multiple groups.

## Results

One hundred forty-nine family doctors responded completely to the questionnaire; 85 were men (57%), 61 were women (40.9%), and the response rate was 0.87%. The average age of the participants was 43.9 ± 10 years, and the average years passed of graduation was 16 years. The duration of experience as a family physician was 6 ± 4 years.

As shown in Tables 1 and 65% of doctors were aware of the DM type 2 national clinical guideline, and 43% had read it.

The average score obtained by the participants was 10 ± 2.4 out of 16 (62.5% of the total score).

The average scores in the screening (3 questions), diagnosis (8 questions), and treatment (5 questions) sections was 1.5 ± 0.8, 5.2 ± 1.6, and 3.3 ± 0.9, respectively. The average score per question in each section was 0.5 ± 0.28, 0.65 ± 0.2, and 0.66 ± 0.17, respectively.

More than 70% of physicians correctly stated the threshold level of FBS to diagnose Diabetes and pre-diabetes, but in the case of HbA1c testing, this rate was less than 50%. Also, most participants know the drug of choice for treating emergency hyperglycemia and the first line of oral medication in DM type-2. The treatment goal was correctly reported in 43%. (Table 2)

Regarding the blood sugar level for the diagnosis of Diabetes, 20% of participants had correctly mentioned the threshold for all three laboratory tests (fasting blood sugar (FBS), HbA1c, and 2-hour post-prandial Blood sugar (2 h-pp BS)).

Eighty-two participants (55%) reported that they would start drug therapy for the fasting blood sugar level of 126 mg/dl, and 18 people (12%) started it at the level of 130 to 150.

As shown in Tables 3 and 57% of physicians requested a creatinine test, and less than 50% requested a lipid profile test for a new case of DM. Only 8% of physicians requested microalbuminuria testing for newly diagnosed diabetes patients.

Sixty-three doctors (42%) had not made any referrals for newly diagnosed uncomplicated diabetic patients. On the other hand, 56 participants (37.6%) would refer these new DM cases to an internist or endocrinologist at the first visit. Referral to ophthalmologists was reported in 49 people (32.89), and referral to a dietician was mentioned in 12 (8.05%).

One hundred forty-two people (95.3%) correctly chose metformin as the first line of treatment.

Regarding the follow-up of a patient with uncontrolled Diabetes who takes oral medication, 68 people (45.6%) correctly mentioned a 2–4 weeks interval would be needed for patients’ follow-up. In comparison, 50 people (33.6%) reported three months or more was required for follow-up intervals.

According to the opinion given by 102 physicians (68.5%), the recommended follow-up interval for patients with controlled DM was every three months.

**Table 1:** The participants characteristics

Characteristics	Type	Frequency (%)
**Sex**	male	85 (57)
	female	61 (40.9)
	missing	3 (2)
**The University from which degree was conferred**	Shiraz	53 (35.6)
	Other	38 (25.4)
	missing	58 (39)
**Employment status**	Private	64 (43)
	Public	68 (45.6)
	Both	12 (8.1)
	missing	5 (3.4)
**Postgraduate education in Diabetes**	Yes	103 (69)
	no	38 (25.5)
	missing	8 (5.5)
**Internet access at workplace**	Yes	122 (82)
	No	23 (15.5)
	missing	4 (2.7)
**Awareness of national guideline**	Yes	97 (65)
	No	48 (32.2)
	missing	4 (2.8)
**Reviewing the national guideline**	Yes	64 (43)
	No	79 (53)
	missing	6 (4)
	**Mean (SD)**	
**Age**	43.9 (10)	
**Years since graduation**	16 (9.5)	
**Duration of experience as a family physician (years)**	6 (4)	
**Average patients/day**	32.3 (18)	

**Table 2:** Knowledge and practice norms of family doctors for screening, diagnosis, treatment, and follow-up of Diabetes type 2

Questions	(%)N	
	incorrect	correct
**Screening**		
1. In adults, if there is no risk factor for Diabetes, at what age do you start screening for Diabetes?	45 (30.2)	103 (69.1) 30 years: 61 (41) 45 years: 42 (28.4)]
2. If the blood sugar screening test was normal, how often do you repeat the screening in adults without any risk factor?	88 (59)	61 (41)
Can HbA1c be used for DM screening?	85 (57)	64 (43)
**Diagnosis**		
3. What level of FBS is defined as “pre-diabetes”?	19 (12.8)	130 (87.2)
4. What 2hpp BS is defined as " impaired glucose tolerance”?	32 (21.5)	117 (78.5)
5. To diagnose pre-diabetes, what is the lower threshold for HbA1c level?	113 (75.8)	35 (23.5)
To diagnose Diabetes, what should be the FBS level?	27 (18.1)	122 (81.9)
6. To diagnose Diabetes, what should be the 2-hrpp BS level?	78 (52.3)	70 (47)
To diagnose Diabetes, what should be the random blood sugar level?	50 (33.6)	99 (66.4)
7. To diagnose DM, what is the lower threshold for the HbA1c level?	84 (56.4)	64 (43)
8. To diagnose DM, how many times do you request FBS?	16 (10.7)	133 (89.3)
**Treatment and follow-up**		
9. Which type of medicine do you usually prescribe in patients with emergency hyperglycemia?	5 (3.4)	144 (96.6)
10. What is the goal of FBS for DM treatment?	84 (56.4)	64 (43)
Do you usually refer a patient recently diagnosed with type 2 diabetes with no other problems?	61 (39)	88 (59)
Which medication category do you most often select as the first-line therapy in managing type 2 diabetes?	7 (4.7)	142 (95.3)
Do you prescribe a second or third antidiabetic drug for patients with uncomplicated type 2 diabetes?	99 (66.4)	50 (33.6)

FBS: Fasting Blood Sugar

2-hrpp BS: 2-hour Post-Prandial Blood Sugar

HbA1c: glycosylated hemoglobin

DM: Diabetes Mellitus

**Table 3:** Laboratory tests requested by family physicians for a new case of diabetes mellitus type 2

Laboratory tests	N	%
Blood urea nitrogen (BUN)	105	70.4698
Creatinine	85	57.04698
High density lipoprotein (HDL)	72	48.32215
Triglyceride	71	47.65101
Low density lipoprotein (LDL)	68	45.63758
Urinalysis (U/A)	62	41.61074
Complete blood count (CBC)	35	23.48993
cholesterol	30	20.13423
alanine transaminase (ALT)	27	18.12081
aspartate transferase (AST)	21	14.09396
Potassium (K)	19	12.75168
Thyroid stimulating hormone (TSH)	16	10.73826
Sodium (Na)	16	10.73826
microalbuminuria	12	8.053691
serum albumin	8	5.369128
Alkaline phosphatase (ALK-P)	3	2.013423
Ferritin, serum Iron, TIBC	3	2.013423
Abdominal sonography	2	1.342282
Serum uric acid	2	1.342282
non	6	4.026846

## Evaluating the related factor

In this study, the effects of 11 factors were evaluated on the total questionnaire score. These factors included age, gender, employment status (public, private), duration since graduation, the average number of patients visited per day, duration of experience as a family physician, postgraduate education about DM, access to the internet in the workplace, awareness of national diabetes guidelines, reading DM national guidelines. Only the " years since graduation " had an independent significant negative relationship with the total score. (Table 4)

**Table 4:** The relationship between years since graduation and the total score of the questionnaires

Variable	B	Std. Error	P-value.
**Years since graduation**	0.06-	0.02	0.003

**Fig. 1:**
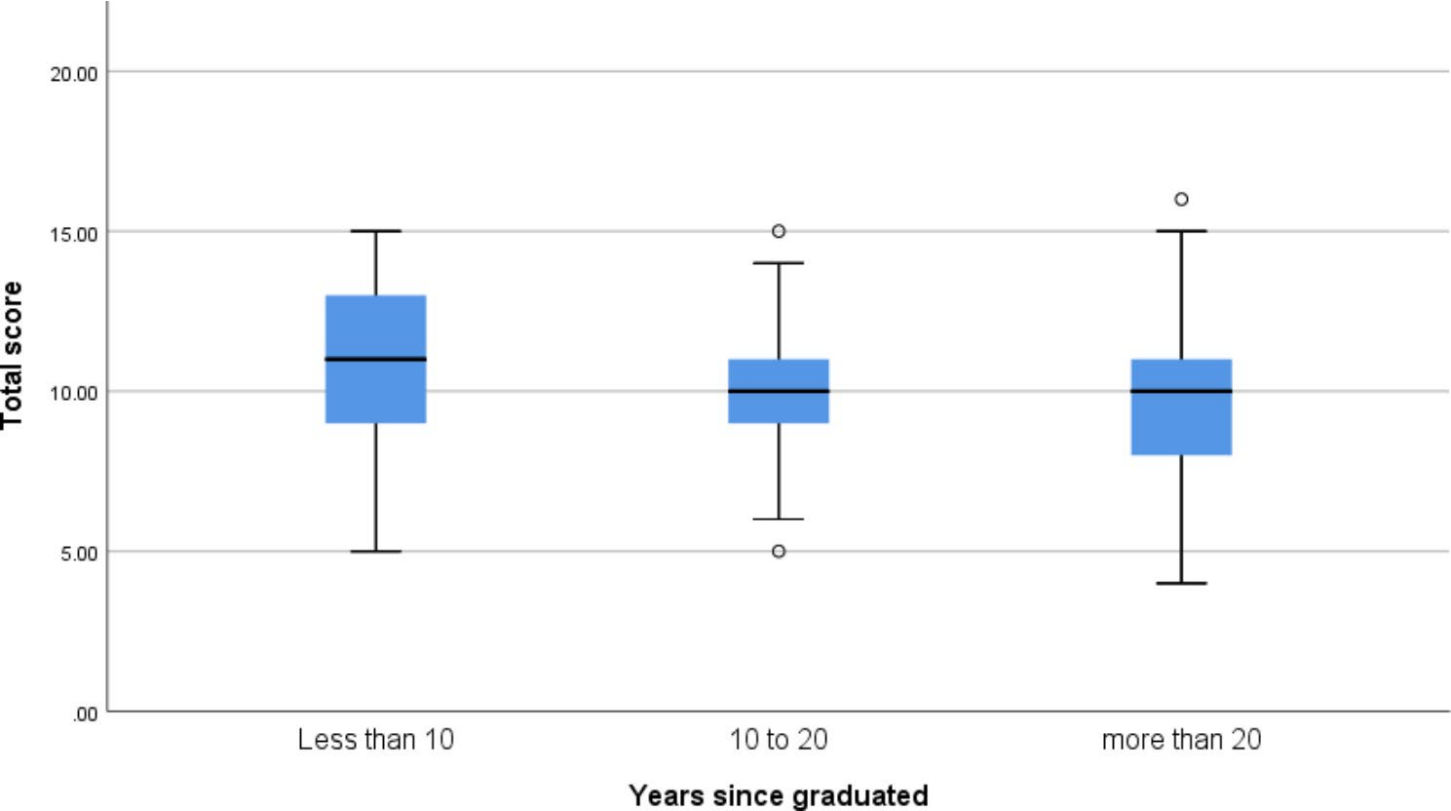
Comparison of the diabetes knowledge questionnaire score among family physicians based on the years since graduation

A comparison was made between three categories of 10-year intervals to better investigate the effect of “duration since graduation” on the questionnaire score. There were 41 (27.5%), 53 (35.6%), and 46 (30.9%) doctors in each group of fewer than ten years,10 to 20 years, and more than 20 years passed from graduation, respectively. As seen in Fig. 1, comparing these three groups showed that their difference was near significant. (F: 2.8, P value: 0.06). Post hoc analysis showed that people who graduated in the last ten years scored significantly better than people who graduated more than 20 years. (P value: 0.03) Also, although the difference between this group and those who graduated 10–20 years prior was not statistically significant, it was very close to significant (P value:0.06).

## Postgraduation preferred methods

**Fig. 2:**
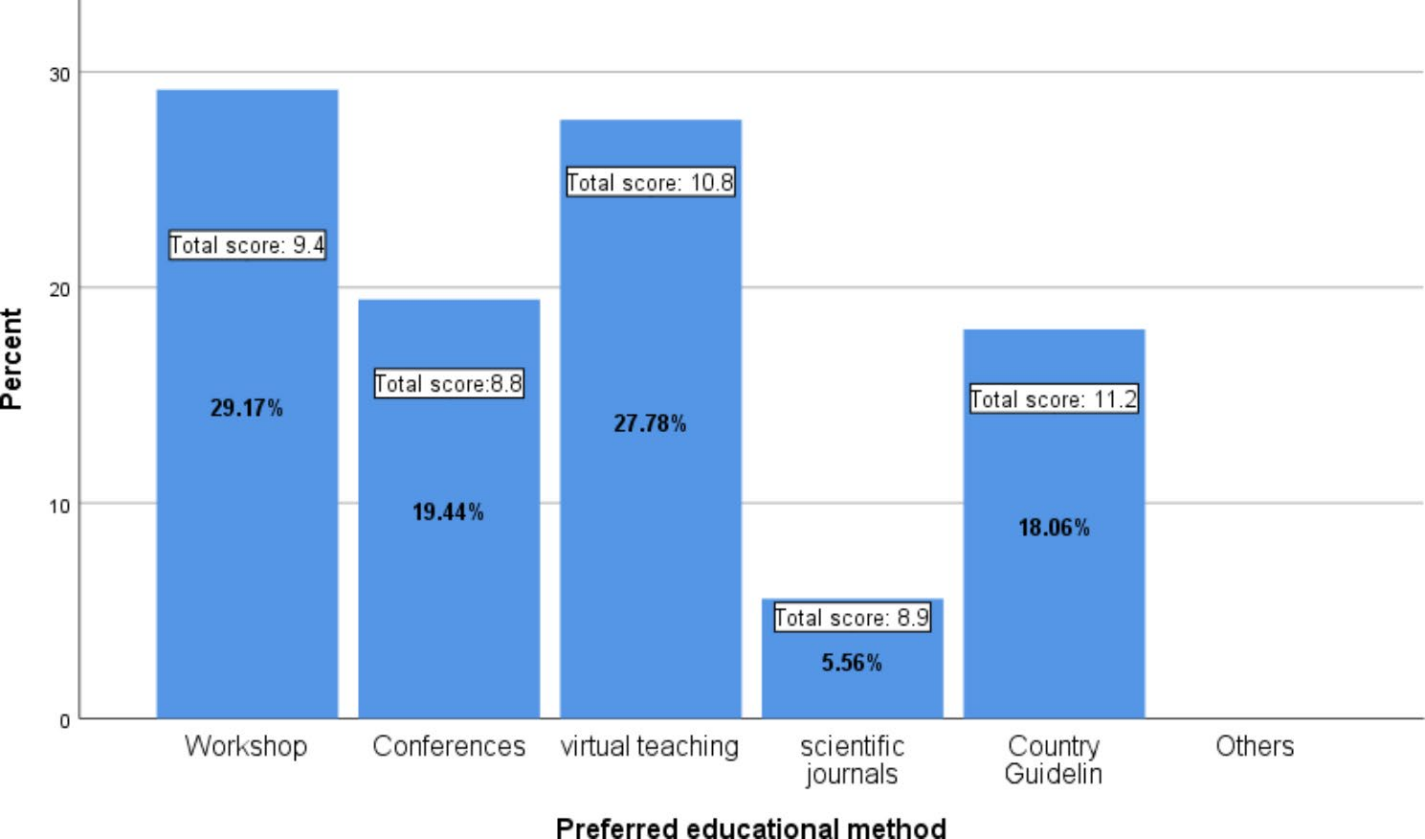
Comparing the percentage of family physicians who chose different educational methods as the preferred method and the total score of the diabetes questionnaire in each group

In response to the preferred method for DM education, most participants preferred the workshop method for training ( Fig. 2), and virtual education was ranked as the second preferred educational method. Conferences, national guidelines, and scientific journals were ranked third to fifth. It should be noted that the people who chose the virtual education method and the use of guidelines have a significantly higher total score on the questionnaire than those who chose the scientific journal or conference. (Table 5)

**Table 5:** Diabetes questionnaire total score differences between groups of physicians with preferred educational methods

	Workshop Mean diff.± SE	Conferences Mean diff.± SE	Virtual teaching Mean diff.± SE	Scientific journals Mean diff.± SE
Conferences	0.5 ± 0.5	0		
Virtual teaching	-1.4 ± 0.5*	-1.9 ± 0.6*	0	
Scientific journals	0.5 ± 0.9	0.02 ± 0.9	1.9 ± 0.9*	0
Country guideline	-1.7 ± 0.6*	-2.3 ± 0.6*	-0.4 ± 0.6	-2.3 ± 0.9*

*P-value < 0.05

## Discussion

The first aim of this study was to evaluate the knowledge and practice norms of urban family physicians in Iran regarding Diabetes management. As the second aim, we tried to find the factors that may affect their knowledge. Also, this study attempted to find the preferred educational method from the participants’ points of view to suggest the most effective educational technique. The study showed that knowledge about screening, diagnosis, and treatment of DM is lower than expected. Most physicians knew the correct threshold of FBS for diagnosing DM and pre-diabetes. They also correctly mentioned the threshold for starting medication and the first line of therapy. Still, they had limited working knowledge about patients’ follow-up intervals and when and to whom they should refer the patients. Only 32% and 8% of doctors mentioned that it is required to refer a new case of Diabetes to an ophthalmologist and a dietician, respectively. Duration since graduation was the only factor affecting the total score of the questionnaire, which had a negative relationship with that.

Although 65% of doctors were aware of the national DM guideline, only 43% had read it. Regarding the preferred educational method from the participant’s point of view, the workshop received the highest preference, and virtual education came second.

In a study in China, 84% of doctors used HbA1c as a diabetes screening test, and this rate was 56% in Saudi Arabia [13]. In our study, 43% of family doctors reported using this test for diabetes screening which was much lower than expected.

The ranges of FBS and 2-hrpp BS and HbA1c have been correctly identified to diagnose pre-diabetes in 87.2%, 78.5%, and 23.5% by family doctors, respectively. In the Nigerian study, participants lacked sufficient knowledge about pre-diabetes diagnosis [8].

In the present study, the percentage of family doctors who correctly knew the threshold values of FBS, 2 h PPBS, Random BS, and HbA1c for diagnosis of Diabetes was 81.9%, 47%, 66.4%, and 43%, respectively. In a study in Cameroon, these numbers were 72.7%, 37.9%, 19.7%, and 32.8%, respectively [15], almost the same as ours. The present study’s results have been better than those in Nigeria, where only 26.6% stated the correct threshold of fasting blood sugar, and about 10% mentioned the threshold of HbA1c [8]. In the mentioned study, only 7.8% of doctors knew all three tests to diagnose pre-diabetes correctly, and 48.4% did not know the range of any of these tests, which in our study, these frequencies were 20% and 8.7%, respectively. A study among primary care physicians in Saudi Arabia showed that more than 80% of the participants had correctly stated the fasting blood sugar and random sugar threshold for diabetes diagnosis. Half of the doctors had correctly displayed the threshold level of the HbA1c test [18], which, compared to our study, indicates a lower level of knowledge in our physicians. This difference may be because general practitioners work in primary care settings in Nigeria, which is very similar to general family physicians in Iran. But in Saudi Arabia, primary care doctors are mostly residents and registrars in specialized training.

On the other hand, better physicians’ knowledge in countries such as China and Saudi Arabia, compared to our country, can be explained by their extensive use of clinical guidelines. In a study in China, it was shown that 83% of doctors were aware of national diabetes guidelines [19] [16], and in Turkey, this rate was 75% [20] [17]. In Saudi Arabia, the level of awareness of the national diabetes guideline was 84.2% [13] [11]. In our country, only 65% of doctors were aware of the national guidelines, and only 43% have read them, which is much lower than expected. It should be noted that reviewing the guideline did not have any significant effect on doctors’ knowledge levels. Our country’s lack of policy to use clinical guidelines in family medicine may be the leading cause of lower-than-expected management outcomes. There is no strategy to inform physicians about guidelines and encourage them to adhere to these evidence-based documents in the family medicine program.

On the other hand, only 22.8% of the doctors in the present study did not consider HbA1c as one of the diagnostic tests for Diabetes. This was a better situation than Nigeria’s study, which reported this rate as 86% [8]. In our study, the number of diagnostic tests needed to diagnose DM in asymptomatic people was correctly mentioned in 89% of cases, which was much better than a similar study in Saudi Arabia [18]. These results indicate that although the usage of guidelines is limited in our country, other methods of obtaining information were helpful in the field of disease diagnosis.

The situation is much worse regarding physicians’ knowledge and practice in screening. Based on different guidelines, the age for starting diabetes screening is different. The ADA guideline recommends screening DM in low-risk adults at 45 years old, but the recommended age for screening is 30 in the national guideline. Although 70% of doctors correctly mentioned the age of starting screening for Diabetes according to any of these guidelines, only 41% answered correctly according to the national guidelines. Repetition of screening intervals and use of HbA1c for screening were mentioned in about 40% of people.

In the present study, about half of the doctors prescribe medication for blood sugar above 126. This result is in harmony with the results of a study in Estonia [21].

Most doctors consider biguanide as the first line of treatment for Diabetes. Still, regarding multi-drug treatment, the goal of treatment in Diabetes, and the referral of new diabetes patients, the knowledge and performance of doctors were poor. In a study in Egypt, treatment modalities were correctly reported by 21–26% of doctors [22]. In a study in Nigeria, the authors concluded that the knowledge and performance of doctors regarding drug treatment in family doctors were weak [8]. In a study in Riyadh, 43–53% of doctors correctly mentioned the treatment goal for Diabetes, which is almost equal to the present study [13]. These results showed that family doctors, regardless of country, seem to have little expertise in treating diabetes cases. It had been shown that family physicians were reluctant to change or add medication for controlling blood sugar. In this way, 55.6% of doctors referred the patients to a diabetologist without changing their drugs when they had uncontrolled Diabetes and had an indication to change their medication [23].

Regarding screening the complications in newly diabetic patients, our study showed, 8%, 57%, and 32.9% of physicians recommended microalbuminuria, serum creatinine measurement, and referral to ophthalmologists, respectively. This statistic is better than Ugwu’s study in Nigeria, where these values ​​were 0%, 23.4%, and 21.9%, respectively [8]. A survey in Switzerland showed that 62% of doctors mentioned annual fundoscopy, and 49% mentioned annual microalbuminuria [24]. We showed that doctors’ performance in our study was much lower than these values. In Amin’s survey in Saudi Arabia, these functions were performed by more than 60% of family doctors [18].

A review study suggested that there was limited research regarding physicians’ knowledge of preventing microvascular complications of DM. However, these studies showed many misunderstandings in follow-up and screening methods of asymptomatic patients to detect microvascular consequences of Diabetes [25].

Regarding the referral of the diabetic patient in our study, 37.6% of the doctors referred newly diagnosed DM cases to internists or endocrinologists, and only 8% referred them to the dietitian. In Fogelman et al.‘s study, 50% of family doctors referred their patients to higher levels of healthcare specialists according to the condition and severity of the disease, and the referral rate for dietitians was more than 94% [9]. This difference is, of course, because in Fogelma’s study, family doctors consisted of 3 categories: family medicine specialists, residents, and physicians who did not have training in family medicine. According to these data, it seems that many general family doctors in our study did not want to manage these patients at the time of diagnosis and prefered to refer them to a secondary level of healthcare. Also, dietitian referral was much lower than in other studies. This problem is mainly due to the guidelines designed for family physicians and the health system’s expectations from primary healthcare providers. The Iranian national DM guideline recommends that any initiation or change of medications, even oral ones, requires referral to specialists. Such recommendations would in turn reduce the family doctor’s responsibility for treating patients and decrease their motivation for treatment.

In the present study, participation in continuing medical education (CME) did not affect the total questionnaire score. Another study in Iran showed that physicians’ knowledge significantly differed after four weeks of online education [26]. It seems that the time intervals between training and evaluation play an essential role in the accuracy of doctors’ responses. This shows the importance of repeating the training at regular intervals. It is necessary to check the continuity of the training effectiveness in different intervals to determine adequate training intervals. A study in Egypt showed that doctors who had an education certification in the DM field or had previously completed relevant training courses had a better score in their informed-knowledge evaluation [22]. This result is entirely contrary to ours, possibly due to the type and quality of education. Another reason may be family doctors’ lack of motivation because, according to national guidelines, they should refer all their new cases to specialists.

A systematic review study investigated the impact of continuing medical education meetings on doctors’ performance. It showed that these training methods could be slightly more effective than no intervention. The factors that could improve the effectiveness of these meetings were the use of other training methods besides the conference, the short duration of the training, low initial performance, follow-up in shorter intervals, and providing other educational materials for studying at home [27]. Lack of effectiveness of continuing education programs in Iran is possibly due to the poor design of these programs, which are usually prolonged and repeated in long intervals.

A study in Saudi Arabia showed that doctors with less experience had more knowledge of diabetes management [28]. This result is consistent with the present study, which showed that the years passed from graduation was the only predicting factor for the total knowledge score. This means that with every year that passed since graduation, the total score of the questionnaire had decreased by 0.06, and people who had less than ten years since graduation had a better score than others. In contrast, the study by Kahf et al. in Egypt showed that doctors who graduated less than five years had a lower score than others [22]. This result may indicate that training methods after graduation in our country are ineffective or that people’s motivation to learn decreases over time.

### Strengths and limitations

As far as we know, this is the first study evaluating general family physicians’ knowledge and practice in Iran. However, as the sampling was done in Shiraz, it isn’t easy to generalize it to the rest of the country covered by the family medicine program. The other limitation is that some participants could not answer the questionnaire due to the workplace crowdedness, which may have caused bias in the results.

## Conclusion

According to the present study, the knowledge and performance of general family doctors about screening, diagnosis, and treatment of DM were much lower than optimal. In the treatment of patients, in the early stages of treatment, such as selecting the first choice of therapy and determining the time to start the medication, most doctors’ knowledge was almost appropriate. Still, in the follow-up and more advanced treatment, like prescribing 2nd or 3rd medications, the knowledge and performance of the doctors were less than expected. Screening for complications was an uncommon practice among physicians. Many participants referred uncomplicated diabetic patients to a secondary healthcare level at the time of diagnosis and did not accept their treatment responsibility themselves. Approximately half of them did not refer newly diagnosed patients at all.

This study also showed that “duration since graduation” was relevant to the physicians’ knowledge. The physicians who graduated in the last ten years had better knowledge than those who graduated more than 20 years ago.

Regarding the preferred method of education, the workshop was the most popular, and virtual training stood in the second position.

### Policy implications

It is necessary to take quick and effective action to increase the awareness and performance of doctors regarding Diabetes management. Implementing the specialty training program for family medicine (which has been started in our country for a few years) is the best final solution. Also, designing a long-term training course for general practitioners can temporarily address this problem. Holding workshops and virtual training are the methods most preferred from the participant’s point of view. So it is suggested that these methods be used as the first training methods. Family physicians are responsible for the follow-up and management of chronic diseases as their primary task, so it is necessary to design a practical and specific guideline for them to enable these practitioners to treat Diabetes in common conditions effectively. Also, it is required to implement a strategy for increasing their desire to adhere to the guideline. It is also necessary to conduct similar studies every 3 to 4 years to check the performance of family doctors and take necessary actions to improve it.

## Data Availability

The datasets used and/or analyzed during the current study are available from the corresponding author upon reasonable request. According to our university regulation, to protect the author’s rights and prevent the information from being used in other articles and systematic reviews before the article is published, this information cannot be made publicly available before publication. But it is available from the corresponding author upon reasonable request.
